# Malaria in Nepal: A Spatiotemporal Study of the Disease Distribution and Challenges on the Path to Elimination

**DOI:** 10.3390/tropicalmed10020046

**Published:** 2025-02-06

**Authors:** Kiran Raj Awasthi, Jonine Jancey, Archie C. A. Clements, Kefyalew Addis Alene, Suman Thapa, Pramin Ghimire, Justine E. Leavy

**Affiliations:** 1 Curtin School of Population Health, Curtin University, Perth, WA 6845, Australia; 2 The Kids Research Institute, Perth, WA 6009, Australia; 3 School of Biological Sciences, Queen’s University of Belfast, Belfast BT7 1NN, UK; 4 Save the Children International in Nepal, Kathmandu 44600, Nepal

**Keywords:** spatiotemporal, malaria, incidence, indigenous, imported, highland

## Abstract

Malaria incidence (MI) has significantly declined in Nepal, and this study aimed to investigate the spatiotemporal distribution and drivers of MI at the ward level. Data for malaria cases were obtained from the National Surveillance System from 2013 to 2021. Data for covariates, including annual mean temperature, annual mean precipitation, and distance to the nearest city, were obtained from publicly available sources. A Bayesian spatial model was used to identify factors associated with the spatial distribution of MI. Between 2013 and 2021, 7278 malaria cases were reported in Nepal, with a crude incidence rate of 3.0 cases per 100,000 person-years at risk (PYR). MI showed a seasonal variation, with the highest number of cases reported between May and September. The annual MI decreased in recent years from 1.9 per 100,000 PYR in 2018 to 0.1 per 100,000 PYR in 2021. Spatial clustering of MI was observed at the ward level, with most hotspot areas detected in the western Terai plains and upper river valley (URV) areas. Incidence was associated with annual mean precipitation in mm (*β* = 0.201; 95% CrI: 0.042, 0.360). The shift of the malaria hotspots to the URVs presents a challenge for implementing timely prevention and control activities.

## 1. Background

Malaria is a vector-borne disease of major public health importance, accounting for an estimated 249 million cases and 608,000 deaths worldwide in 2021 [1,2]. While 95% of malaria cases occur on the African continent, 2% of the remaining malaria cases are clustered in the World Health Organization Southeast Asia Region (WHO-SEAR), with the major contributor being India (65% of the regional cases) [1]. *Plasmodium vivax (P. vixax)* and *Plasmodium falciparum* (*P. falciparum*) are the two predominant species of parasite causing malaria in the SEAR. The SEAR represents 54% of the global *P. vivax* cases, of which 47% are found in India alone [2].

Nepal’s efforts to eliminate malaria are significantly challenged by its open border with India [3]. Frequent and uncontrolled migration of Nepali seasonal workers to highly malaria-endemic regions in India, combined with inadequate testing and screening facilities at border crossings, facilitates the continued transmission of malaria within Nepal [3,4]. Malaria cases in Nepal are classified as either indigenous or imported. According to the Nepal National Malaria Program (NMP), indigenous malaria refers to infection acquired within the country by individuals with no travel history, whereas imported malaria refers to cases contracted abroad and diagnosed in Nepal [5]. In 2021, 83.5% of the malaria cases in Nepal were classified as imported, primarily affecting migrant workers returning from India [6].

Although Nepal has successfully reduced the number of malaria cases by 90%, from 12,750 cases in 2002 to 447 cases in 2020, 41.3% of the population still are at risk [6,7]. Interventions such as free distribution of long-lasting insecticidal nets (LLINs), indoor residual spraying (IRS), prompt diagnosis and treatment of cases with antimalarial drugs including Artemisinin-based Combination Therapy (ACT) to treat *P. falciparum* malaria, and focused prevention and control activities have all contributed to the decline in case numbers [8]. Of the two variants found in Nepal in 2021, 91.7% of cases were caused by *P. vivax* while the remaining were *P. falciparum* infections [6,9]. Recent malaria data show a higher disease burden in the western parts of Nepal, with 83% of the total malaria cases reported from three western provinces (Sudurpaschim, Karnali, and Lumbini Provinces) [6]. Malaria in Nepal predominantly occurs in lowland areas with a subtropical/tropical climate, high temperatures, abundant rainfall, and year-round cultivation, a combination of factors that suits vector survival [10]. However, in recent years, malaria cases have been reported from the upper river valley (URV) regions of Nepal, the valleys between the hills and mountains (areas over 2000 m above sea level) [6,9,11,12]. In 2018 and 2019, the URV contributed to 20% and 28% of the reported total malaria cases in Nepal, respectively [11,13,14].

Nepal aims to eliminate malaria by 2025 [15]. Like other vector-borne diseases in a pre-elimination and elimination period, malaria cases often present a focal spatial distribution, with certain geographically defined hotspots having a comparatively higher caseload than the surrounding areas [16]. Climate, ecology, and human factors drive the spatiotemporal variation in malaria risk. Information on spatial patterns and related drivers can assist the NMP in devising cost-effective interventions to control and eliminate malaria in high-risk areas [17]. For example, knowledge of the spatiotemporal distribution of malaria hotspots will allow the NMP to predict areas at risk of transmission and epidemics, providing data to initiate early prevention measures [18].

Spatial epidemiological investigations have been conducted previously to characterise and explain the spatial distribution of malaria in Nepal; however, they have been focused on areas of the country rather than the whole country [19,20,21,22]. Recently, Bhattarai et al. conducted a nationwide study on the spatiotemporal distribution and drivers in Nepal at the district level (2013 to 2021) based on the pre-federalisation administrative boundaries [9,23]. Nepal adopted a federal structure in 2015 with new administrative boundaries. This study investigated the spatiotemporal distribution and drivers of malaria transmission at the ward level (the lowest administrative level) using a Bayesian spatial analysis for both main species of malaria parasite (*P. vivax* and *P. falciparum)* and types of cases (imported and indigenous) between 2013 and 2021 [9]. This period, therefore, encompasses major milestones such as federalisation (2015), ward-level malaria microstratification (2016, 2018) [24,25], and the pre-elimination phase based on the National Malaria Strategic Plan (NMSP) 2014–2025 [15]. As such, this study adds further detailed evidence and complements the existing literature.

## 2. Methods

### 2.1. Study Area

**Figure 1:**
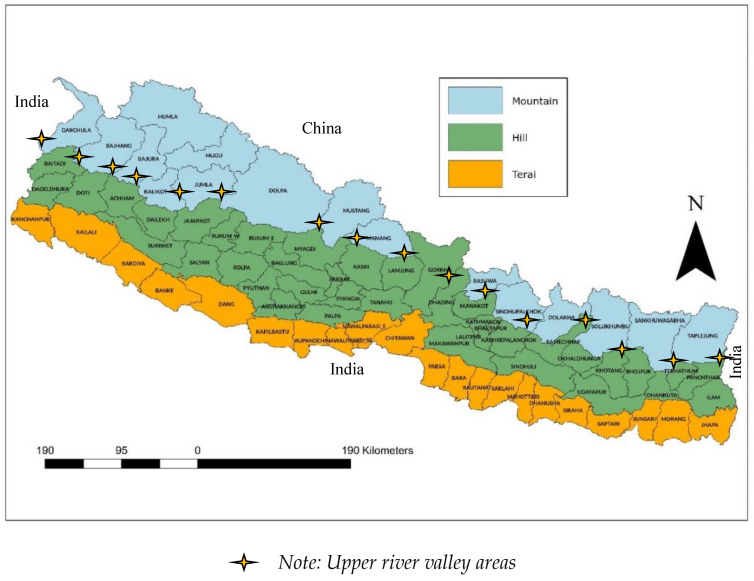
Geo-administrative map of Nepal showing the Terai plains, middle hills, and upper mountains along with the 77 districts.

Nepal is a landlocked country bordering China to the north and India to the east, south, and west, and shares an 888 km open border with India along the southern belt [11]. The country is spread over 147,516 square kilometres and has a population of 28.8 million [26,27]. Administratively, the country is divided into seven provinces, 77 districts, 754 municipalities, and 6743 wards (the smallest administrative units) [28]. Geographically, Nepal is divided longitudinally into three parts: the plain Terai (altitude < 610 m); middle hills (altitude 610–4800 m); and high mountains (altitude 4800–8848 m) (Figure 1). Nepal shows an extreme topological variation in elevation, ranging from 59 m above sea level in the south to 8848 m above sea level in the northern Himalayas. Many human settlements are found in the Terai and valleys in the hills and mountains due to the rich water supply from streams and rivers that make these areas ideal for agriculture. The country experiences four seasons: winter (December–February); spring (March–May); summer (June–August); and autumn (September–November). The peak rainfall occurs in July during the monsoon.

### 2.2. Malaria Surveillance Data

The Health Management Information Section (HMIS) and Epidemiology and Disease Control Division (EDCD), under the Department of Health Services (DoHS), are responsible for maintaining the data of the national malaria surveillance program. HMIS and EDCD work closely with the provincial and municipal health units to obtain malaria data in three forms: immediate phone message notification of malaria cases through the Malaria Disease Information System (MDIS); weekly notification of cases diagnosed at 118 sentinel sites through the Early Warning and Reporting System (public and private hospitals and medical colleges, mostly at the district level) [28]; and the monthly reporting of cases through the integrated HMIS [6]. The information received from the three sources is collated, cross-verified, and cleaned by the two departments, before being published in the yearly DoHS Annual report. Malaria diagnosis in Nepal is mainly performed through a WHO pre-qualified Rapid Diagnostic Test (RDT) on symptomatic people. All RDT-positive cases are re-confirmed microscopically by trained microscopists at the nearest public health facilities. Reactive case detection is carried out by a joint EDCD and the local municipality team for each confirmed case to identify additional cases, which are subsequently reported to the HMIS. All information on confirmed cases is maintained at multiple tiers: the municipality; district; province; and country. This study includes monthly data from the national reporting system for malaria cases of both types (indigenous and imported) and species (*P. vivax* and *P. falciparum*), reported between January 2013 and December 2021.

### 2.3. Climate, Elevation, and Population Data

Data for environmental and climatic covariates were obtained from publicly available sources (Supplementary Table S1). A polygon shape file of the administrative boundaries at statistical areas (level 3) for Nepal was obtained from the DoHS and Global Administrative Areas (GADM). Raster maps of average rainfall and temperature were obtained from the Worldclim website [29,30]. Data on distance to cities (i.e., walking travel times in minutes to the nearest cities) were obtained from the Malaria Atlas Project (MAP) [31]. Altitude measurements of the elevation of the earth’s land surface in metres were obtained from the Shuttle Radar Topography Mission (SRTM) [32]. The national census data of 2011 and the World Bank annual population growth estimate rate were used to compute the monthly population counts of wards between January 2012 and December 2021 [26,27]. The independent variables were selected based on evidence of association with malaria infection from previous studies and the availability of high-resolution raster files for the study settings [1,2,3,4]. All covariates were geo-referenced and linked to the dependent variable (malaria cases) using a geographical information system (GIS; ESRI Inc., Redlands, CA, USA).

### 2.4. Statistical Analysis

#### 2.4.1. Crude Malaria Incidence Rate

As a descriptive analysis, the crude malaria incidence rate (CMIR) was calculated by year. The CMIR was calculated by dividing the total number of new malaria cases by the population of the same year in the corresponding ward, multiplied by 100,000 to obtain a rate per 100,000 population. The CMIR was described and compared according to species and type of malaria. The annual incidence of malaria was analysed and described for all nine years (2013 to 2021) for total, indigenous, imported, *P. vivax*, and *P. falciparum* cases.

#### 2.4.2. Standardised Incidence Ratio

Standardised incidence ratios (SIRs) were calculated for each ward and year. For each ward i, i =1…n, the SIR was calculated as the ratio of the observed number of malaria cases in the ward ($Y_{i}$) to the expected number of malaria cases ($E_{i}$) in the ward across the study period:

$$SIR_{i}=Y_{i}/E_{i}$$

The expected count $E_{i}$ represented the total number of malaria cases that one would expect if the population of the ward i had the same risk as the national population. The expected number of malaria cases for each ward ($E_{i}$) was computed as

$$E_{i}=r_{j}^{\left(s\right)}n_{j}^{\left(s\right)},$$

where $r_{j}^{\left(s\right)}$ is the CMIR for Nepal (i.e., the total number of malaria cases divided by the total population in all wards), and $n_{j}^{\left(s\right)}$ is the population of each ward i.

#### 2.4.3. Spatial Autocorrelation Analysis

The global Moran’s I and Getis–Ord statistics were used to identify clusters of high MI at the ward level across all regions of Nepal. The global Moran’s I statistic was used to assess the presence, strength, and direction of spatial autocorrelation and to test the assumption of spatial independence in implementing the spatial pattern analysis [33]. The Getis–Ord statistic was used to detect the local clustering of malaria infection [34]. Maps produced from the Moran’s I and Getis–Ord statistics show the existence of malaria infection clusters and identify the locations of potential hotspot areas.

#### 2.4.4. Bayesian Spatial Analysis

Although SIRs can be useful in some settings, in wards with small populations, the expected counts are very low, and SIRs may be insufficiently reliable for reporting. Therefore, we also estimated malaria infection risk using Bayesian spatial models that enable estimates to borrow information from neighbouring wards and incorporate information from covariates, resulting in the smoothing or shrinking of extreme values in areas with small populations.

The model was constructed using covariates, unstructured random effects, and spatially structured random effects. The observed numbers of malaria cases $Y_{i}$ in ward i, were modelled using a Poisson ($P_{o})\;$ distribution with mean $E_{i}\theta_{i}$, where $E_{i}$ is the expected number of malaria cases and $\theta_{i}$ is the relative risk in ward i. The logarithm of the relative risk $\theta_{i}$ was expressed as the sum of an intercept that models the overall risk level of malaria, a vector of covariates and their coefficients, and random effects to account for extra-Poisson variability. The model for the spatial data is expressed as follows:

$$Y_{i}\;\sim \;P_{o}\left(E_{i}\theta_{i}\right),\;i=1,\ldots ,\;n$$

$$\log(\theta_{i})=a+\beta d_{i}+u_{i}+v_{i}$$

Here, a represents the overall risk of malaria in the country, $\beta d_{i}$= (1, $\beta d_{i1},\ldots ,\beta d_{ip})$ is the vector of p covariates corresponding to ward i, and $\beta =(\beta_{0},\;\beta_{1},\;\ldots ,\beta_{p}$) is the coefficient vector of the covariates. In this setting, for a one-unit increase in covariate $\beta d_{j\;},\;j=1,\ldots ,p$, the relative risk increases by a factor of ${\exp\beta }_{j}$, holding all other covariates constant. Additionally, $u_{i}$ is a random effect specific to ward i to model spatial dependence between the relative risks, and $v_{i}$ is an unstructured exchangeable component that models uncorrelated noise, $v_{i}\;\sim \;N(0,\;\sigma_{v}^{2}$).

The spatial random effect $u_{i}$ was assigned a Conditional Autoregressive (CAR) distribution using a queen neighbourhood weight matrix, which specified that two areas are neighbours if they share a common boundary. Specifically,

$$u_{i}/u_{\_\_i}\;\sim \;N({ŭ}_{\sigma i},\;\frac{\sigma_{u}^{2}}{n_{\sigma i}})$$

where ${ŭ}_{\sigma i}=n_{\sigma i}^{-1}\sum_{j\in \sigma i}u_{j},\sigma i\;$ and $n_{\sigma i}$ represent, respectively, the set of neighbours and the number of neighbours of ward i. The unstructured component $v_{i}$ was modelled as independent and identically distributed normal variables with zero mean and variance $\sigma_{v}^{2}$. Non-informative priors were specified for the intercept α (a non-informative, improper prior with bounds − ∞ and + ∞) and the coefficients (normal prior with mean = 0 and precision 1 × 10^−6^). The priors for the precision of the unstructured and spatially structured random effects were assigned non-informative gamma distributions with shape and scale parameters set at 0.001. A relatively large number of samples (20,000 samples) was computed to ensure that a satisfactory characterisation of the posterior distribution of all parameters could be obtained. The posterior parameters were estimated using the Integrated Nested Laplace Approximation (INLA) approach in R statistical software (R-INLA, version 4.0.2) (R Core Team). The maps were created using ArcGIS version 10.6.1 (ESRI Inc.; Redlands, CA, USA).

Before the model was fitted, all covariates were checked for multi-collinearity using Pearson correlation coefficients. Those variables with a high correlation coefficient were excluded from the final model. Distance to the nearest city, annual mean temperature, and annual mean precipitation were eligible covariates to be included in the final model (Supplementary Table S1). Since these independent variables had different units and scales of measurement that would have unknown threshold effects, the variables were normalised using their mean and standard deviation ([X-mean]/SD). This method also helped identify the estimation of the posterior distribution of the coefficients. The final model was selected using the Watanabe–Akaike information criterion (WAIC) statistics, and multiple models were tested with different components in the process.

## 3. Results

### 3.1. Descriptive Analysis

**Figure 2:**
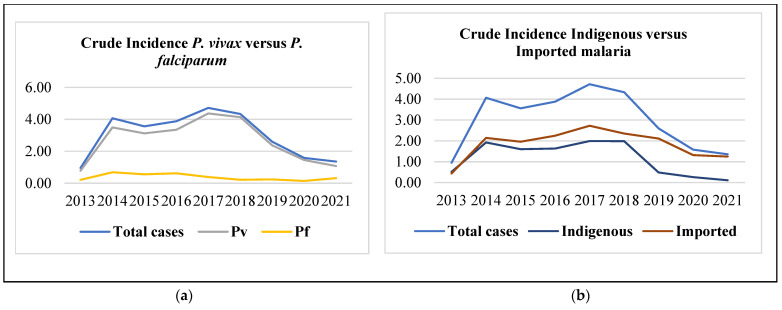
(**a**,**b**). Crude incidence of malaria per 100,000 risk years in Nepal between 2013 and 2021.

In total, 7278 malaria cases were reported in Nepal between 2013 and 2021, with a crude incidence of 2.98 cases per 100,000 person-years at risk (PYR). During the nine years, 6376 *P. vivax*, 780 *P. falciparum*, and 122 mixed infections (both *P. vivax* and *P. falciparum*) were reported. Between the two variants, a higher incidence of *P. vivax* was observed over *P. falciparum* (2.61 versus 0.32 cases per 100,000 PYR). Across the study period, 2805 indigenous and 4473 imported cases were reported, with a crude incidence of 1.15 and 1.83 cases per 100,000 PYR, respectively. The crude incidence was high for *P. vivax* cases between 2016 and 2018 and sharply declined afterward (Figure 2a).

A sharp increase in crude incidence for both indigenous and imported cases was noted in 2014 and 2017. Interestingly, a steep decline in crude incidence was observed between 2018 and 2021, notably more for indigenous cases, which decreased from 1.9 in 2018 to 0.1 cases per 100,000 PYR in 2021. A gradual decline in the crude incidence of imported cases was also evident during the same period (2.4 and 1.3 cases per 100,000 years at risk) (Figure 2b).

**Figure 3:**
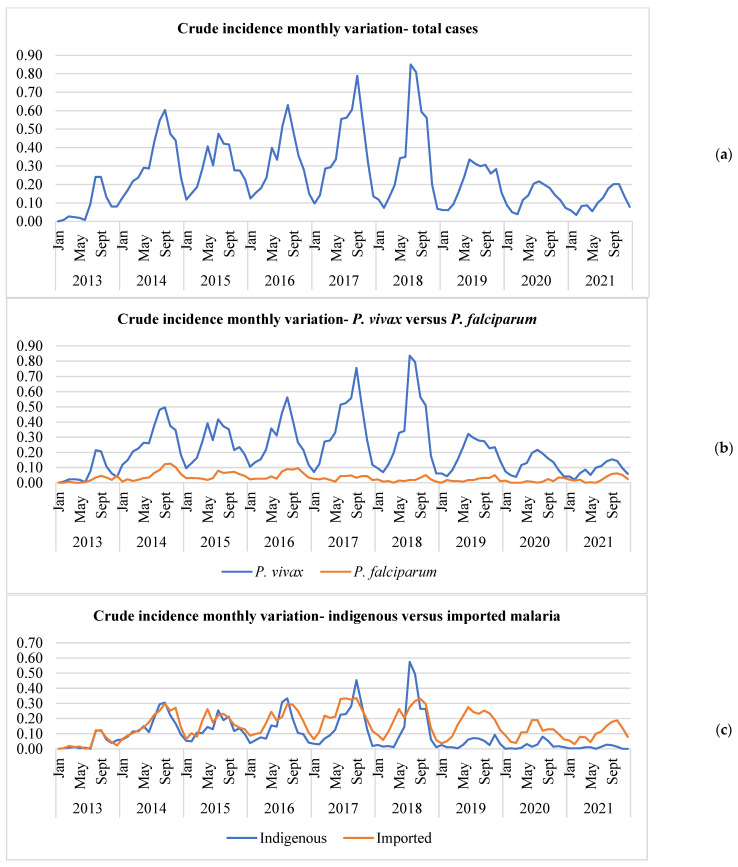
(**a**–**c**) The monthly incidence of malaria per 100,000 risk years in Nepal between 2013 and 2021 for total cases (**a**), *P. vivax* vs. *P. falciparum* (**b**), and imported vs. indigenous (**c**).

MI in Nepal varied seasonally, with higher incidence reported during the summer (May to September) each year (Figure 3a). Malaria cases increased from May and peaked during July–August and then declined in October. Similar seasonal patterns of incidence were observed for both species and types (Figure 3b,c). Malaria incidence was very low during the winter (November to February).

The MI map of Nepal between 2013 and 2021 showed a higher incidence of the disease in the Terai region. Malaria cases were dispersed east to west along the southern border with India; however, a higher incidence was observed in the western region of Nepal compared to the central and the eastern regions. Notably, a high incidence of indigenous and imported *P. vivax* malaria was also located in some URV areas in the west (Supplementary Figure S1).

**Figure 4:**
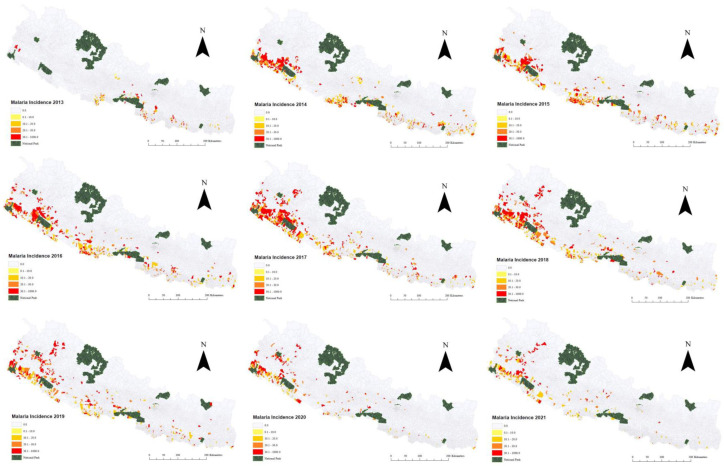
Annual crude incidence of malaria cases per 100,000 person-years at risk (2013–2021).

Due to low case numbers, cumulative MI maps (2013–2021) of total, *P. vivax*, *P. falciparum*, indigenous, and imported cases were created to understand their spatial distribution (Supplementary Figure S1). The separate yearly maps showed a distinct shift of MI towards western Nepal after 2016 (Figure 4). Between 2016 and 2021, a higher MI was observed in the three western provinces (Lumbini, Karnali, and Sudurpaschim) of Nepal. Increased incidence of malaria in the URV was observed from 2015 onwards, mostly in two provinces (Karnali and Sudurpaschim). The malaria clusters in the URV were situated along the corridors of the Karnali river (Bajura, Mugu, and Humla districts) that separates Karnali and Sudurpaschim Provinces and the Mahakali river (Dadeldhura, Baitadi) separating Sudurpaschim Province and India (Supplementary Figure S2: file map showing Karnali and the Mahakali river along with the seven provinces).

### 3.2. Spatiotemporal Distribution of Malaria

**Figure 5:**
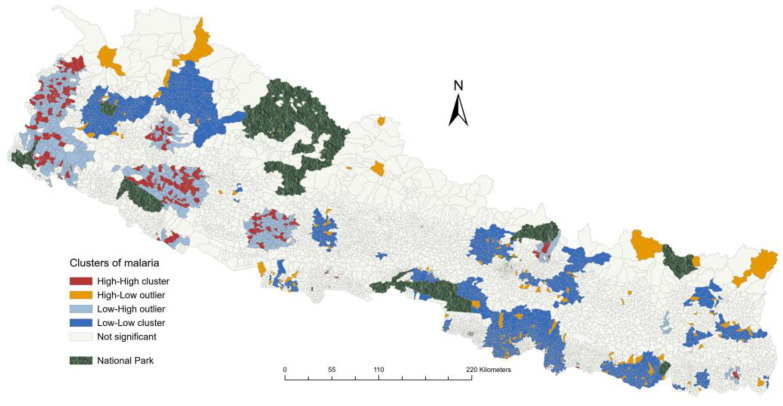
Map of Nepal showing ward-level risk clusters of malaria between 2013 and 2021 using Moran’s I statistics.

**Figure 6:**
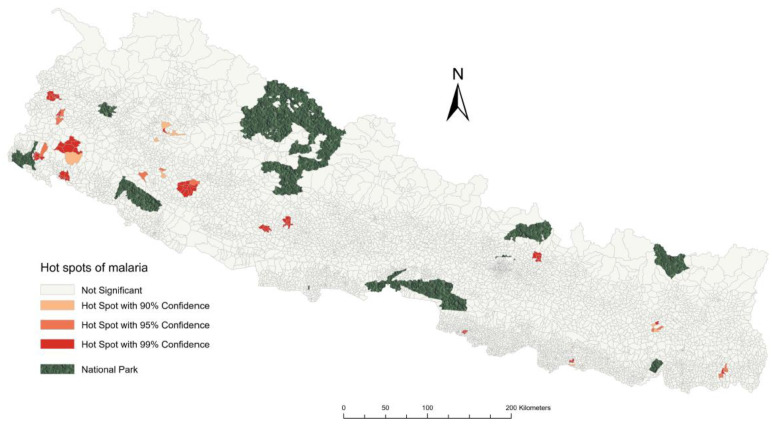
Spatial distribution of malaria at the ward level in Nepal and the hotspot clusters between 2013 and 2021 using Getis–Ord analysis.

The spatial clustering analysis identified malaria risk areas with high–high clusters predominantly observed in the western parts of Nepal across Lumbini, Karnali, and Sudurpaschim Provinces (Figure 5). In Sudurpaschim Province, these clusters were observed in Kailali, Kanchanpur, Dadeldhura, Baitadi, and Darchula districts. However, the high–high clusters were mostly found in wards along the Mahakali river bordering India in the URV areas of Dadeldhura, Baitadi, and Darchula. Similar clusters were found in Surkhet, Kalikot, and Dailekh districts of Karnali Province and Banke district of Lumbini Province. A single cluster was found in Sindhupalchowk district of Bagmati Province and Jhapa district of Koshi Province. The Getis–Ord analysis showed sparsely scattered hotspots in thirteen districts across six provinces; however, the largest number of hotspots was found in Sudurpaschim Province (Figure 6). All the hotspot wards were present in either the lower plains or middle hills of the country.

**Figure 7:**
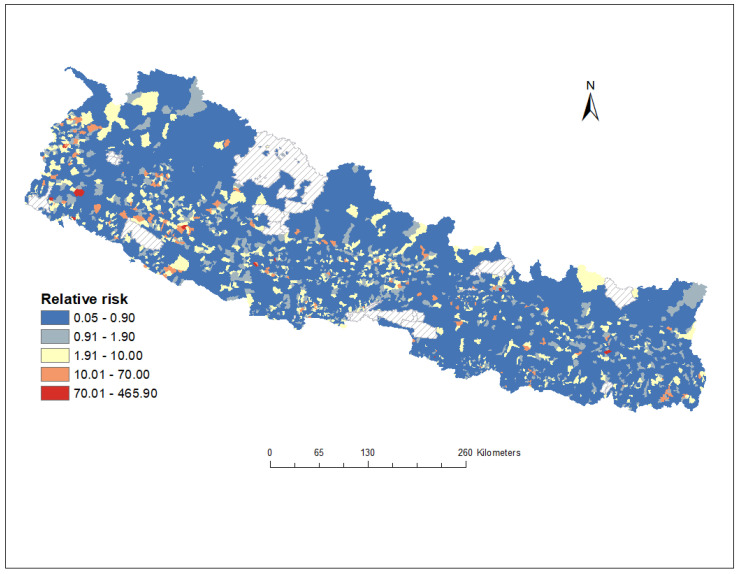
Relative risk (RR) of malaria infection at ward level in Nepal based on the posterior spatially structured random effects, 2013–2021. The RR quantified whether a ward has a higher (RR > 1) or lower (RR < 1) risk than the average risk in the state population. For example, if (RR = 2), this meant that the risk for the ward is two times the average risk in the country’s population. The grey cross-hatch areas on the map are national parks and wildlife reserves.

Table 1 shows the Bayesian multivariable Poisson regression model of ecological-level factors associated with the incidence of malaria in Nepal. Based on having the lowest WAIC value, the model contains both structured and unstructured spatial random effects in the best-fitting model. The model showed that the incidence of malaria cases was associated with annual mean precipitation in mm (*β =* 0.201; 95% CrIa: 0.042, 0.360). After accounting for the ecological-level factors in the model, the posterior mean of the spatially structured random effects revealed clusters of residual risk in the country. This indicates that a substantial amount of ward-level heterogeneity in malaria remained unexplained by the ecological-level factors included in our models (Figure 7).

## 4. Discussion

Nepal aims to eliminate malaria by the end of 2025 [15]. Similar to its neighbouring countries in the South Asia region such as Bhutan, Bangladesh, and Pakistan, our results also show a gradual decrease in MI in Nepal between 2013 and 2021 [35,36,37]. Recently, some high-malaria-risk states in India have started to show a significant decline in MI; this is an important factor to consider in Nepal’s decline in malaria cases as India is the main source of imported malaria in Nepal [3,36,38]. Scaling up of LLINs and periodic IRS in high- and moderate-risk areas of the country, improved surveillance, early diagnosis, and treatment of malaria cases as per the national treatment protocol could have contributed to the decline in malaria cases in Nepal [5,6]. Vector control activities in Nepal include biannual IRS conducted by the NMP in high- and moderate-malaria-risk wards, and in areas identified as active foci as a part of the foci investigation and response strategy. Likewise, mass distribution of LLINs is conducted every three years in high- and moderate-risk wards, according to the most recent microstratification. Both of these activities have played a crucial role in the decrease in MI in Nepal [6]. However, since the malaria cases in the region are co-existent and associated, the NMPs in WHO_SEAR, including Nepal, need an agreed strategy to identify and treat imported cases early, thereby preventing local transmission of the disease.

The spatial analysis revealed that the wards at an increased risk of malaria were not uniformly distributed throughout the country. The high–high-malaria-risk wards and hotspots were distributed in specific parts of the western Terai (lowland) region, bordering India, and situated in the river valley areas of the hills and mountains (referred to as the URV), including highland areas at an altitude of more than 2000 m above sea level. Between 2016 and 2019, our results showed a high MI in the highlands of five districts: Humla and Mugu in Karnali Province; and Baitadi, Bajura, and Dadeldhura in Sudurpashcim Province. Malaria presence in both highland and lowland areas warrants further exploration and focus from the NMP. Lowland malaria is common worldwide due to factors that include cultivation, dense forests, and subtropical climates in these regions [10,17]. However, global warming and climate change [39], rapid urbanisation with road access to remote areas [40,41], vector adaptation [42], and constant movement of people have shifted malaria risk areas to the highlands in many countries across Africa and Asia [43,44,45,46,47]. Highland malaria has been reported in WHO-SEAR countries such as Afghanistan and Bhutan, with the latter being close to malaria elimination [35,48]. Highland malaria cannot be neglected as it can easily derail a country’s elimination efforts; however, managing malaria in the highlands is challenging due to their remoteness, treacherous geographical terrains, and limited road access [49]. Hence, information about the shifts in the distribution of malaria hotspots is crucial for Nepal’s NMP when allocating resources and planning interventions.

Notably, since 2019, we found a decrease in the total MI and a distinct difference between the two types of malaria in Nepal. In recent years, malaria was found to have shifted to the western part of the country. Indigenous MI in Nepal showed a steeper decline compared to imported malaria post-2019. Several factors could have contributed to this finding. The indigenous cases could have declined due to the regular interventions conducted in four active malaria foci in Karnali (Mugu and Humla districts) and Sudur Paschim (Baitadi and Bajura) Provinces. These four wards accounted for 197 and 342 indigenous cases of malaria in 2017 and 2018 [11,50] which decreased to 44 and 18 cases, respectively, in 2019 and 2020, suggesting an impact of the concentrated control efforts of the NMP [6]. The most probable explanation for the relatively slower decline in imported malaria is the continued seasonal migration of the Nepalese population to and from malaria-endemic locations in India as they search for work [3]. In the Karnali and Sudurpaschim Provinces, the poverty levels are much lower compared with the other five provinces [51]. As poverty is associated with poorer living standards and unaffordable preventive measures, this may explain the higher burden of malaria in these two provinces. An exploration of the recent nationwide migration patterns of the Nepalese population to understand the shift in MI to the western region would be worthwhile. This would provide the NMP with crucial information to plan preventive strategies for the mobile migrant population based on the risk status. India has been a hub for malaria infection in the region and is the main contributor to imported malaria in neighbouring countries, including Nepal, Bhutan, and Bangladesh [21,49,52]. India contributes 79% of the total malaria cases and 83% of deaths due to malaria in Southeast Asia [2]. An estimated 1.5 million Nepalese from low- and middle-income households migrate to India annually, a historical trend in existence since the 18th century [4]. Seasonal migrants living and working in high-risk environments (particularly farms) and in high-risk occupations, such as army personnel, security guards, and factory workers, are most at risk [3,4,53]. Hence, cross-border malaria control strategies such as inter-country collaboration, mass media campaigns, and easy access to adequately resourced health facilities along high-malaria-risk bordering districts to carry out intensified surveillance activities should be a priority [54]. Such strategies have been successfully deployed by the national malaria programs of Thailand, Myanmar, and Laos, and can be replicated along the Indo–Nepal border [54,55].

Factors such as temperature, precipitation, altitude, and distance to nearby cities (relating to health care access) are predictors of malaria risk population and malaria transmission [12,19]. These predictors can shed light on and explain the spatiotemporal distribution of and seasonal variation in the disease. Seasonal variation is quite common for malaria, with higher numbers of cases being reported during the summer-monsoon period in South and Southeast Asia, a time when vector populations peak [17]. July and August in Nepal coincide with the monsoon; however, cases are still observed until late October. It is believed that optimal rainfall extends the longevity of mosquitoes in high-risk areas, thereby prolonging the transmission lag time [18]. Several studies have confirmed that the lag pattern between malaria transmission and rainfall shows a heterogenous association, both in hot districts in the lowlands and in cold districts in the highlands [18,45]. However, the lag times vary from two to five months based on the temperature, as reported by a vector study conducted in Sri Lanka [56]. A major festival in Nepal falls during September and October, when most Nepalese migrants return home from India, potentially aiding disease transmission [13]. This lag could be a future challenge for the NMP due to continued transmission even in early winter.

Finally, the COVID-19 pandemic could also have indirectly contributed to the decline in overall reported numbers of malaria cases in Nepal post-2019. COVID-19 has similar symptoms to malaria; however, malaria testing rates post-COVID-19 decreased in Nepal from 252,156 tests in 2019 to 156,788 in 2020, possibly resulting in lower case detection rates [50,57]. In 2020, during the COVID-19 pandemic, The Global Fund (the major funding partner for Nepal’s NMP) reported a disruption in 73% of malaria programs across 100 countries, mostly due to delayed distribution of LLINs and IRS and test kits, along with health and laboratory staff being reassigned to combat COVID-19 [57]. Staff shortages in the NMP’s prevention and control programs impacted the implementation of elimination efforts in multiple countries, with Nepal being no exception [58]. Hesitation to be tested due to fear of contracting COVID-19 at public health facilities and delayed diagnosis and treatment can enable malaria outbreaks, further stressing the health system [2,59]. In such situations, recruiting emergency volunteers and staff to conduct routine surveillance, testing, and treating of diagnosed malaria or even mass drug administration (MDA) could be a temporary solution for the NMP [60].

This study analysed a reasonably long time series (nine years) of malaria data at a high spatial resolution (ward level) in Nepal, a strength of the study. Crucially, this period included the malaria control, pre-elimination, and elimination phases and the COVID-19 pandemic, thus providing information on the latter’s effect on MI. The new federal administrative boundaries were considered during the analysis. However, this study has several limitations. The analysis included rainfall, altitude, temperature, and distance to the nearest city as covariates for which data were easily available. Other potentially important environmental factors such as the normalised difference vegetation index, surface water, entomological factors, and population mobility were not included due to incomplete data [18]. In recent years, municipalities have focused on opening new transportation routes by opening new tracks and building roads even in remote URV areas [44]. This along with other factors such as mobility, seasonal migration, and habits of staying outdoors without appropriate clothes could have played a role in the occurrence of malaria hotspots; however, there is limited availability of such data, and hence they could not be included. Only passive surveillance data were used in the analysis, and as such, the dataset only included cases captured from the public sector and major private hospitals. Records from local unregistered private service providers (local pharmacies and clinics) are not captured by the national base database, which might have contributed to underreporting [6,15]. Finally, projected population data were used for each ward, which may have resulted in under- or overestimation of the incidence.

## 5. Conclusions

MI in Nepal has declined in the last two decades. Despite making notable progress on the path to elimination, the spatiotemporal analysis shows a heavy concentration of active hotspots in the western part of Nepal. The recent shift of the malaria hotspots to the highlands in the URVs is of concern to Nepal’s NMP, as it could be challenging to conduct early preventive and control activities and initiate outbreak responses. Although the decline in indigenous malaria cases is positive, the ever-present risk of imported malaria through constant movement of populations to and from these hotspots, particularly from malaria-endemic areas in neighbouring India, will be a challenge in Nepal for the foreseeable future.

## Figures and Tables

**Table 1:** Bayesian Poisson regression models of *P. vivax* and *P. falciparum* malaria, Nepal, 2013–2021.

Variables	Model I: Fixed Effect Only	Model II: Unstructured	Model III: Structured	Model IV: Structured and Unstructured
	Coefficient, Posterior Mean (95% CrI)	Coefficient, Posterior Mean (95% CrI)	Coefficient, Posterior Mean (95% CrI)	Coefficient, Posterior Mean (95% CrI)
Annual mean temperature (°C)	−0.035 (−0.073, 0.003)	0.046 (−0.066, 0.159)	−0.075 (−0.393, 0.244)	−0.035 (−0.246, 0.176)
Annual mean precipitation (mm)	0.101 (0.074, 0.129)	0.222 (0.141, 0.303)	0.160 (−0.108, 0.428)	0.201 (0.042, 0.360)
Distance to city (minutes)	0.044 (0.004, 0.085)	0.151 (0.030, 0.270)	0.025 (−0.232, 0.279)	0.018 (−0.174, 0.209)
α (intercept)	−0.003 (−0.028, 0.022)	−2.148 (−2.271, −2.035)	−2.186 (−2.302, −2.082)	−2.131 (−2.249, −2.022)
WAIC	32,679.32	10,950.74	13,451.19	10,756.30

CrI: credible intervals.

## Data Availability

The original contributions presented in this study are included in the article/Supplementary Materials. Further inquiries can be directed to the corresponding author.

## Supplementary Materials

The following supporting information can be downloaded at: https://www.mdpi.com/article/10.3390/tropicalmed10020046/s1, Figure S1: Maps showing malaria incidence for the period of 2013–2021 in Nepal; Figure S2: Map of Nepal showing the major rivers and seven provinces; Table S1: Data sources and definitions of covariates.
