# Supplementary material for: Malaria in Nepal: A Spatiotemporal Study of the Disease Distribution and Challenges on the Path to Elimination
Source: Trop Med Infect Dis. 2025 Feb 6;10(2):46. doi: 10.3390/tropicalmed10020046 (PMC11860284; doi:10.3390/tropicalmed10020046)
Supplement: Supplementary file 1 [file tropicalmed-10-00046-s001.zip › tropicalmed-3372971-supplementary.pdf]

Supplementary File: Malaria in Nepal: A spatiotemporal study of the disease distribution in Nepal and the challenges to elimination

Kiran Raj Awasthi,[a,] Jonine Jancey,[a] Archie C. A. Clements,[b,c] Kefyalew Addis Alene,[a,b] Suman Thapa,[e] Prakash Shah,[d] Pramin Ghimire,[d] and Justine E. Leavy[a]*

[a]Curtin School of Population Health, Curtin University, GPO Box U1987, Perth, WA 6845, Australia
[b]Telethon Kids Institute, Perth, Western Australia, Australia

[c]Peninsula Medical School, University of Plymouth, Plymouth, United Kingdom

[d]Epidemiology and Disease Control Division, Department of Health Services, Nepal

[e]Save the Children International in Nepal, Nepal

*** Correspondence**: kiran.awasthi@curtin.edu.au; Tel.: +61-424906590

Supplementary Table 1: Data sources and definitions of covariates

Covariates	Data sources	Definitions
Temperature	WorldClime	Annual mean environmental air temperature (°C) (1)
Precipitation	WorldClime	Annual mean rainfall (mm) (1)
Altitude	Shuttle Radar Topography Mission (SRTM)	Elevation of the earth land surface in meter (2)
Distance to the nearest cities	Malaria Atlas Project (MAP)	Walking travel times in minutes to the nearest city (3)
Nepal polygon shapefile	Global Administrative Areas (GADM)	Administrative boundaries at Statistical Areas Level (3)

Description: Table 1 describes the data sources and the defines the covariates used in the analysis.

Supplementary Figure 1. Maps showing malaria incidence for the period of 2013-2021 in Nepal

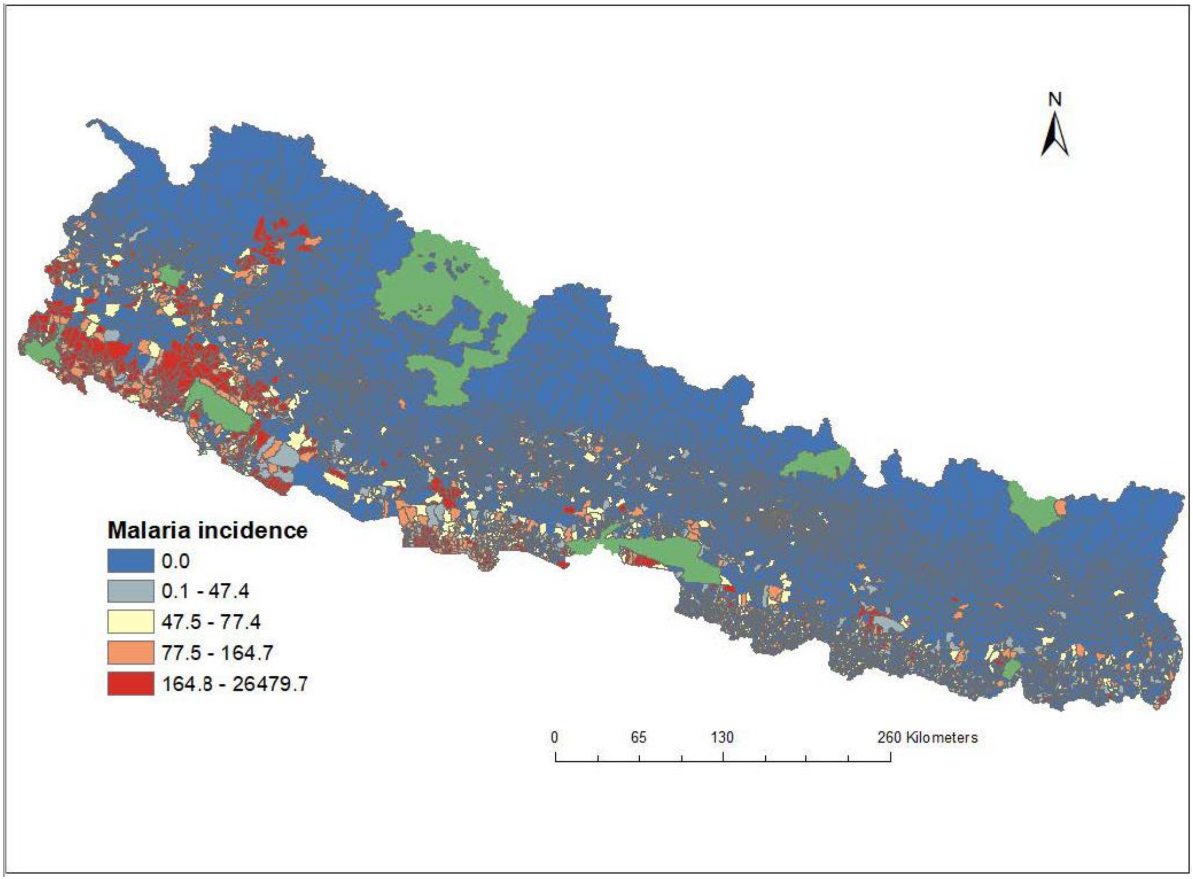

a

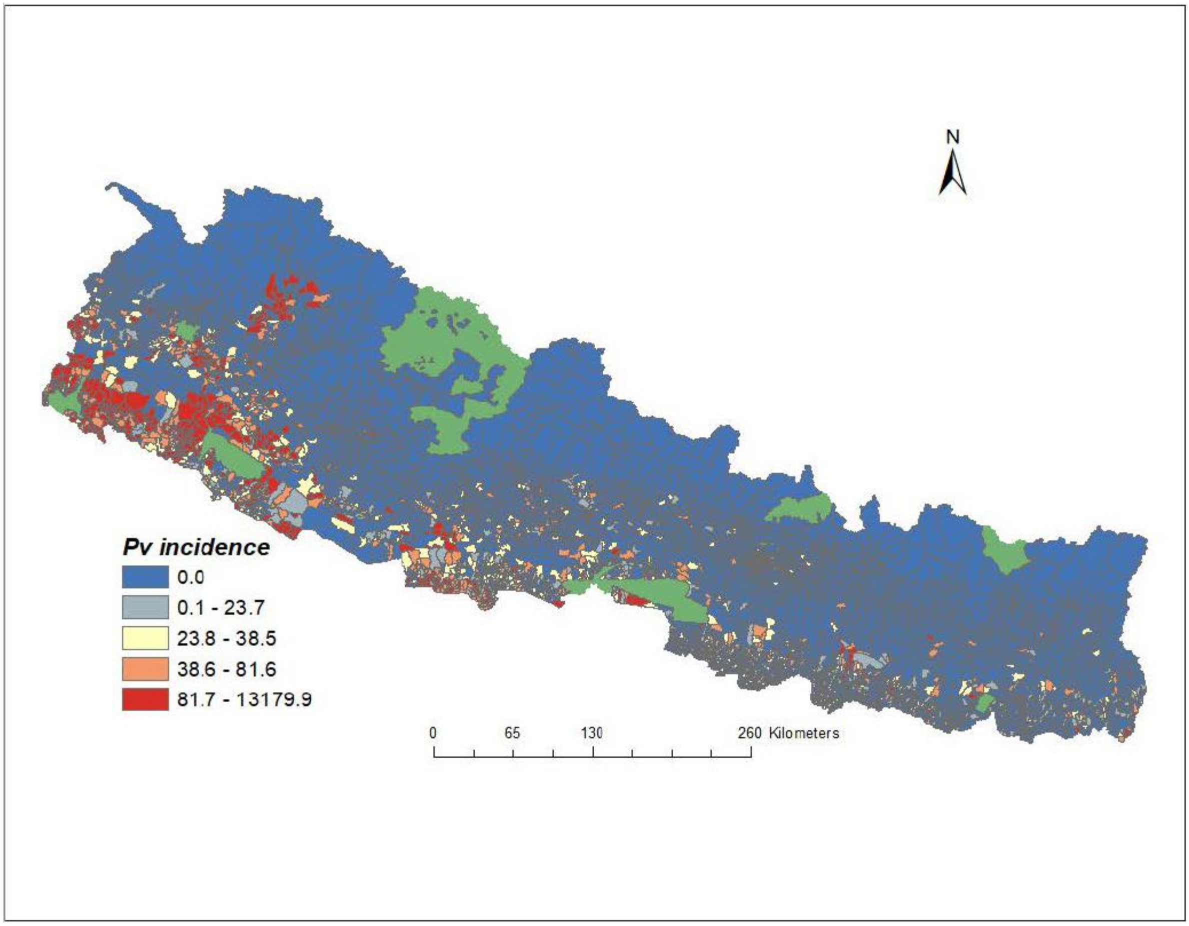

b

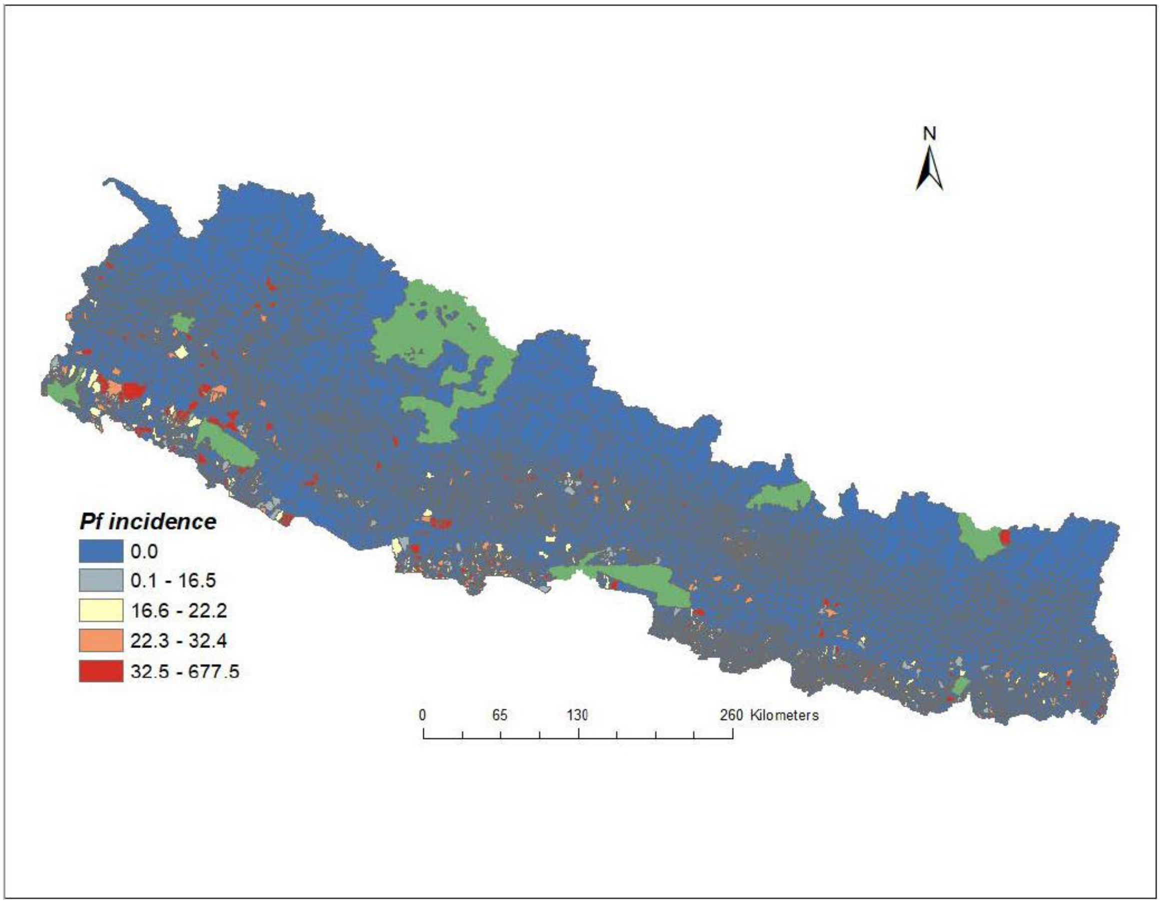
N
Pf incidence
0.0
0.1 - 16.5
16.6 - 22.2
22.3 - 32.4
32.5 - 677.5
0
65
130
260 Kilometers
c

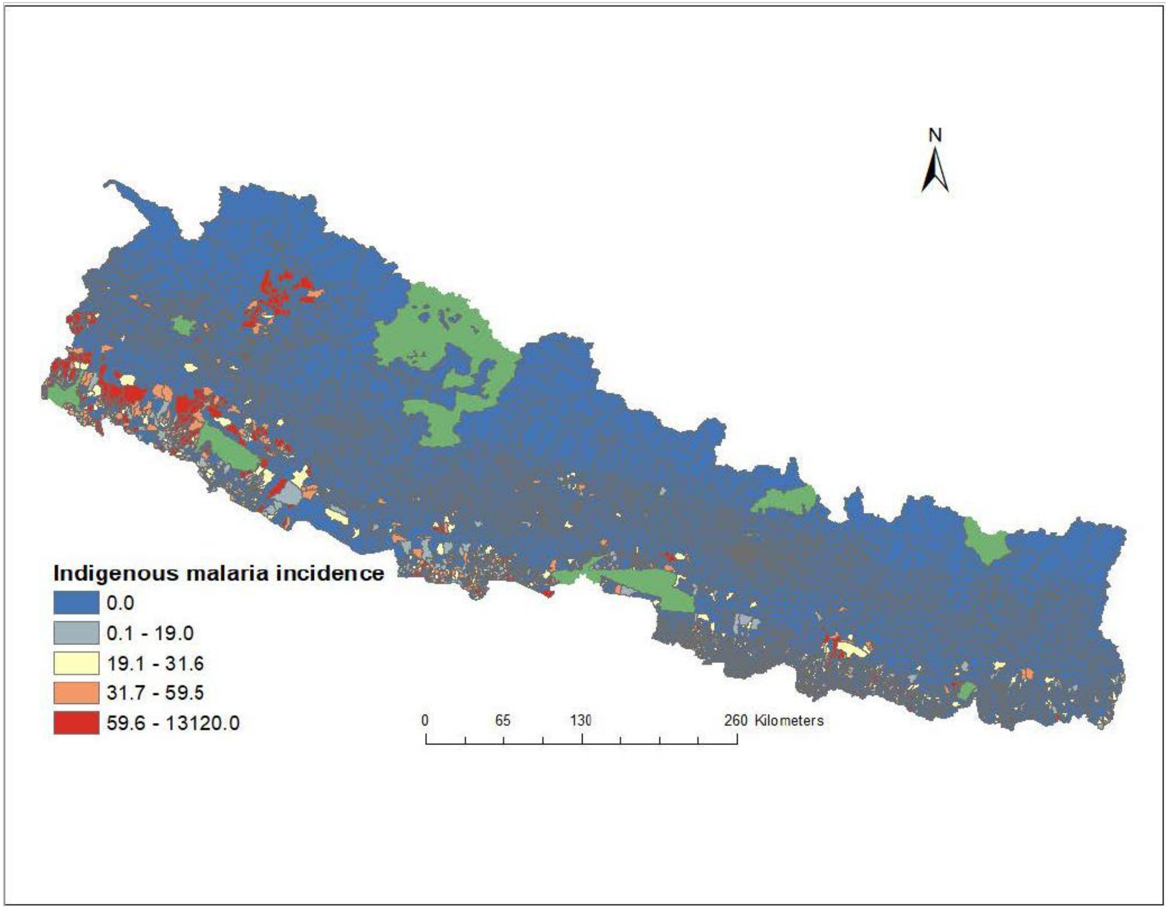
N
Indigenous malaria incidence
0.0
0.1 - 19.0
19.1 - 31.6
31.7 - 59.5
59.6 - 13120.0
0
65
130
260 Kilometers
d

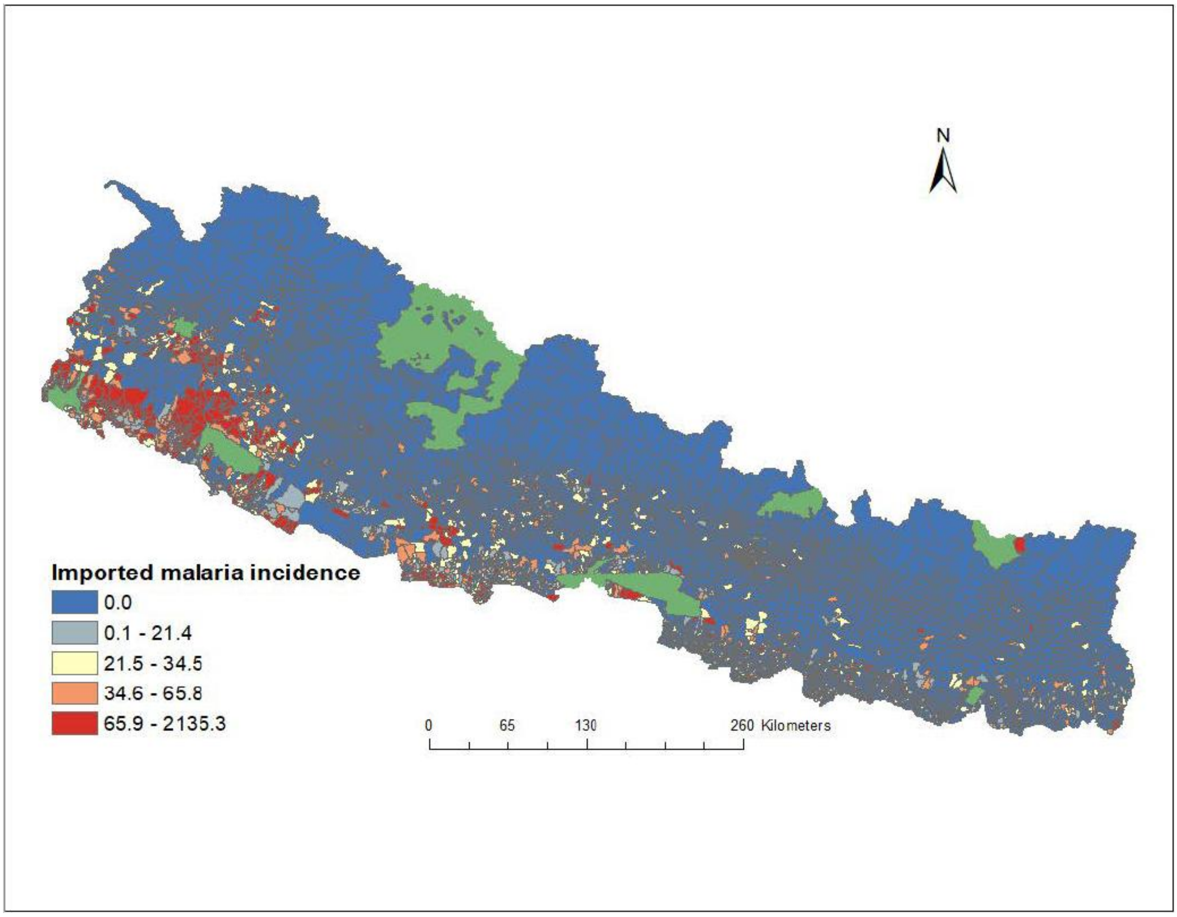

e

Description: Figure 1 shows the cumulative malaria incidence map for the nine years (2013-2021) for the overall malaria (a), based on species- P.*vivax* and *P.falciparum* (b, c) and types- imported and indigenous (d, e)

Supplementary Figure 2. Map of Nepal showing the major rivers and seven provinces

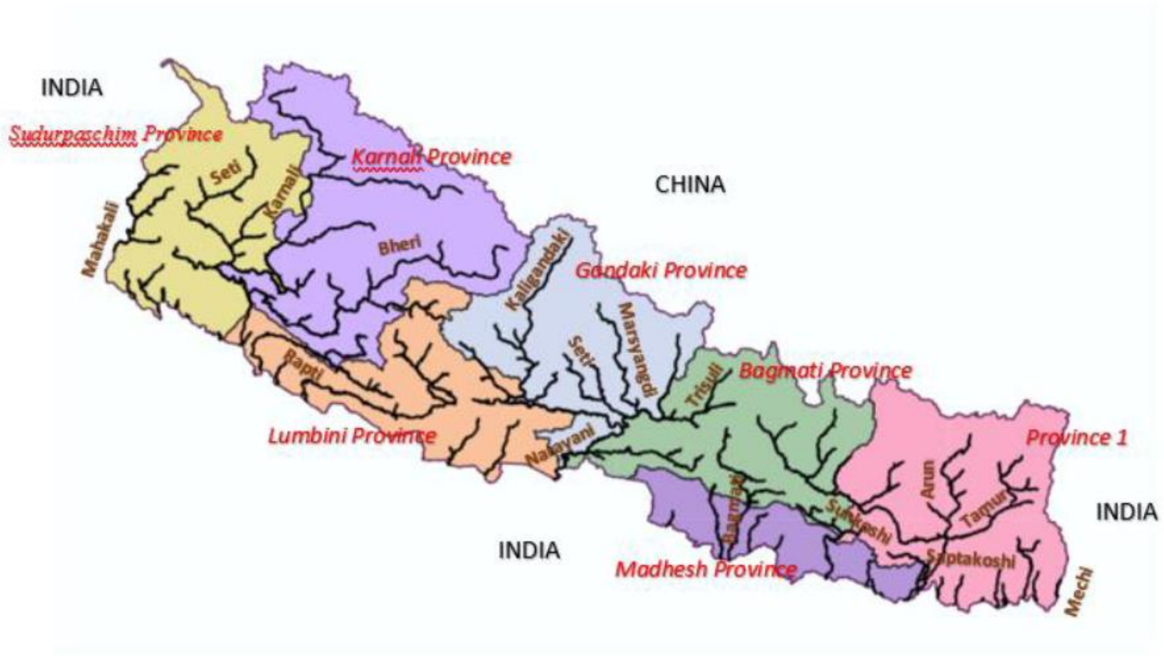

Description: The map above shows the seven provinces of Nepal and the major rivers in the country. The main rivers of interest are Karnali (which separates Karnali from Sudurpaschim Province) and Mahakali (which separates Sudurpaschim Province from India) along which a higher incidence of malaria is observed. Despite many rivers in the central and eastern region of the country, yearly incidence maps do not show any cases around them.
